# *Odoribacter splanchnicus* elicits lung protection via vesicle-driven enhancement of the host Cav1–Ces1d interaction

**DOI:** 10.3389/fmicb.2026.1860203

**Published:** 2026-07-07

**Authors:** Qixin Yang, Zeliang Wu, Kangjie Ding, Xuanyu Gu, Rumeng Mei, Meng Shao, Feilong Chen, Cuiping Jiang, Weili Han, Qingfa Tang

**Affiliations:** 1 School of Traditional Chinese Medicine, Southern Medical University, Guangzhou, China; 2 Guangdong Provincial Key Laboratory of Chinese Medicine Pharmaceutics, Guangzhou, China; 3 School of Public Health, Southern Medical University, Guangzhou, China

**Keywords:** acute lung injury, Cav1–Ces1d interaction, extracellular vesicles, gut–lung axis, *Odoribacter splanchnicus*

## Abstract

Dysregulated inflammation and barrier dysfunction are central features of acute lung injury (ALI). Mounting evidence underscores the gut–lung axis as a critical pathway in pulmonary inflammation, yet how specific commensal bacteria confer distal organ protection remains unclear. Here, we demonstrate that the gut commensal *Odoribacter splanchnicus* elicits marked protection against lipopolysaccharide-induced acute lung injury (ALI) in mice, primarily through its extracellular vesicles (O-EVs). Depletion of *O. splanchnicus* exacerbated pulmonary damage and inflammatory cytokine release, whereas restoration of its abundance or administration of purified O-EVs significantly attenuated lung injury. Integrated transcriptomic and proteomic analyses identified caveolin (Cav1) and carboxylesterase 1d (Ces1d) as critical host targets of O-EVs. We found that O-EVs were associated with enhanced Cav1–Ces1d interaction, which correlated with suppressed activation of NF-κB and STAT3 signaling and decreased levels of downstream pro-inflammatory mediators (TNF-α, IL-1β, IL-6, iNOS, SOCS3). Concurrently, O-EVs reduced leukotriene B4 (LTB4) production, indicating restraint of Ces1d-associated lipid inflammatory pathways. Lipidomic profiling revealed that O-EVs are enriched in bacterial sphingolipids and anionic phospholipids with immunomodulatory potential. Collectively, these data indicate that the gut bacterium *O. splanchnicus* acts as a key regulator of gut–lung axis communication, mediating anti-inflammatory protection in acute lung injury through vesicle-dependent modulation of inflammatory signaling, lipid mediators, and immune responses.

## Introduction

Acute lung injury (ALI) and its severe complication, acute respiratory distress syndrome (ARDS), are debilitating syndromes marked by uncontrolled inflammation, epithelial and endothelial barrier disruption, and impaired gas exchange (Zhang et al., 2024; Zhou et al., 2025). Despite advances in supportive care, mortality remains high, emphasizing the need for new therapeutic strategies (Ma et al., 2025). Increasing evidence indicates that ALI is closely linked to systemic immune responses and inter-organ communication (Wang et al., 2025; Ziaka and Exadaktylos, 2024), with the gut–lung axis emerging as a key regulatory pathway whereby microbial metabolites and molecules modulate pulmonary immunity and injury progression (Ren et al., 2024; He et al., 2024). Perturbations in gut microbiota (dysbiosis) exacerbate lung inflammation, whereas restoring microbial balance mitigates pathology (Popovic et al., 2023; Tang et al., 2024). High-resolution multi-omics studies further reveal that specific microbial taxa and their secreted factors can reprogram immune and metabolic pathways in the lung, highlighting the gut–lung axis as a promising target for ALI intervention (Lv et al., 2023; Shen et al., 2023; Hu et al., 2024).

Within the diverse gut ecosystem, *Odoribacter splanchnicus*, a Gram-negative commensal bacterium, has gained increasing attention as a beneficial microbial species. Belonging to the *Bacteroidetes* phylum, *O. splanchnicus* is enriched in healthy individuals but often depleted in patients with chronic inflammatory and metabolic diseases (Hiippala et al., 2020; Li et al., 2025). Its abundance is positively associated with anti-inflammatory metabolic profiles, including the production of short-chain fatty acids and bile acid derivatives, which are known to attenuate systemic inflammation (Li et al., 2025). Recent metagenomic studies have highlighted *O. splanchnicus* as a key species maintaining gut barrier integrity and immunological tolerance, with correlations to improved outcomes in inflammatory bowel disease and metabolic syndrome (Lv et al., 2023; Lima et al., 2022). Intriguingly, alterations in *O. splanchnicus* abundance have also been observed in patients with pulmonary disorders, further suggesting its potential involvement in gut–lung crosstalk (Zheng et al., 2021; Lima et al., 2022). However, the precise mechanisms by which *O. splanchnicus* exerts protective effects beyond the gut remain poorly defined. Recent advances indicate that vesicle-mediated communication may be a central modality through which this bacterium influences distant organs, including the lungs.

Bacterial extracellular vesicles have recently emerged as potent mediators of microbiota–host communication. These nanoscale, bilayered vesicles encapsulate proteins, lipids, nucleic acids, and metabolites, enabling bacteria to influence host cells in a highly targeted and stable manner (Zheng et al., 2021; Diaz-Garrido et al., 2021; Yi et al., 2025). Compared with soluble metabolites, bacterial extracellular vesicles have unique advantages: they are structurally protected from enzymatic degradation, capable of crossing biological barriers, and exhibit natural tropism toward specific host tissues (Huang et al., 2023; Tian et al., 2023). Recent high-impact studies have demonstrated that gut microbiota-derived vesicles can regulate immune cell polarization, modulate epithelial tight junctions, and mitigate inflammatory responses in distant organs including the lungs (Tao et al., 2025; Hu et al., 2024; Kumar et al., 2024). For instance, bacterial extracellular vesicles from commensal *Bacteroides* species have been shown to promote anti-inflammatory pathways and confer protection against systemic inflammatory challenges (Fonseca et al., 2022). In the context of ALI, bacterial extracellular vesicles represent an attractive therapeutic modality, offering the ability to deliver bacterial signals without the risks associated with live bacterial administration. However, most studies to date have focused on generic microbiota-derived vesicles, while the role of bacterial extracellular vesicles derived from *O. splanchnicus* (O-EVs) remains largely unexplored.

Caveolin-1 (Cav1), a principal structural component of caveolae, plays a critical role in maintaining endothelial barrier integrity and orchestrating inflammatory signaling in the lung. Dysregulation of Cav1 has been associated with increased vascular permeability, excessive activation of NF-κB- and STAT3-related inflammatory pathways, and aggravated inflammatory injury during acute lung injury (ALI) (Maniatis et al., 2012; Fan et al., 2024). In parallel, carboxylesterase 1d (Ces1d), a lipid-hydrolyzing enzyme involved in intracellular lipid metabolism, has recently been recognized as an important modulator of inflammatory responses through its regulation of lipid-derived inflammatory mediators and cellular metabolic homeostasis (Szafran et al., 2022). Alterations in Ces1d activity have been linked to abnormal lipid signaling and sustained production of pro-inflammatory cytokines, highlighting a close connection between lipid metabolism and inflammatory control. Although Cav1 and Ces1d have traditionally been investigated as independent regulators of membrane signaling and lipid metabolism, respectively, accumulating evidence suggests that coordinated interactions between membrane scaffolding proteins and lipid-metabolizing enzymes may critically shape host inflammatory responses under stress conditions (Luo et al., 2021). However, whether and how Cav1 and Ces1d functionally interact to jointly regulate inflammatory signaling and lipid-driven inflammation in ALI remains largely unexplored.

We hypothesize that the gut commensal *O. splanchnicus* exerts distal protective effects in acute lung injury by modulating the functional interaction between Cav1 and Ces1d through vesicle-mediated signaling. By strengthening the Cav1–Ces1d axis, O-EVs is proposed to coordinately regulate endothelial barrier integrity and inflammatory responses, including suppression of NF-κB- and STAT3-dependent inflammatory signaling and attenuation of lipid-derived inflammatory mediator production. Elucidating this bacterium-driven regulatory framework provides mechanistic insight into how gut microbiota communicate with the lung and confer protection against inflammatory lung injury via extracellular vesicles.

## Materials and methods

### Animals

All animal experimental protocols were conducted in compliance with institutional ethical standards and approved by the Animal Management and Use Committee of Southern Medical University (Review Number: SMUL202312027) and the Institute of Biological and Medical Engineering, Guangdong Academy of Sciences (Review Number: K2024-01-124). Wild-type C57BL/6 mice (5 weeks old) were purchased from a specific pathogen-free (SPF) facility. Animals were housed in a sterile environment under controlled conditions (temperature 20–22 °C, relative humidity 50 ± 2%, 12 h light/dark cycle).

### Animal experiments

The ALI model was established by intranasal administration of lipopolysaccharide (LPS, 5 mg/kg) using a microsyringe for two consecutive days (Soliman et al., 2018). The appearance of moist rales in the lungs was considered an indicator of successful model establishment.

In the fecal microbiota transplantation (FMT) experiment, male C57BL/6 mice (5 weeks old) were administered a combination of antibiotics—vancomycin (100 mg/kg), neomycin sulfate (200 mg/kg), metronidazole (200 mg/kg), and ampicillin (200 mg/kg)—for five consecutive days to deplete gut microbiota. Following antibiotic pretreatment, the pseudo-germ-free mice were randomly divided into two groups and orally administered 0.2 mL of fecal suspension supernatant (0.125 g/mL) daily for 7 days. The fecal material was obtained from either healthy donors (FMT-CON) or lipopolysaccharide (LPS)-induced acute lung injury mice (FMT-MOD).

For the pharmacodynamic study of *O. splanchnicus*, male C57BL/6 mice (5 weeks old) were randomly assigned to four groups: control (CON), model (MOD), live *O. splanchnicus* (OS), and heat-killed *O. splanchnicus* (OS-HK). The CON group received sterile PBS intranasally for two consecutive days. Following model induction, the CON and MOD groups were administered 0.2 mL of sterile PBS daily, while the OS and OS-HK groups received equal volumes of live or heat-killed *O. splanchnicus* suspensions (3 × 10^8^ CFU/mL), respectively. The doses used in both the OS group and the OS-HK group were based on the plate colony counts prior to inactivation. All treatments were continued for seven consecutive days.

For the pharmacodynamic study of O-EVs, male C57BL/6 mice (5 weeks old) were divided to four groups: control (CON), model (MOD), low-dose (EVs-L), and high-dose (EVs-H). The ALI model was established as described above. After modeling, the CON and MOD groups received 0.2 mL of sterile PBS daily, whereas the EVs-L and EVs-H groups were administered O-EVs at doses of 10 μg/day and 20 μg/day in 200 μL PBS, respectively. The doses of O-EVs refers to the total protein content in the vesicular preparation. All treatments were continued for seven consecutive days.

### Cultivation of *Odoribacter splanchnicus*

*O. splanchnicus* glycerol stock (1 mL) was inoculated into 50 mL Reinforced Clostridial Medium (RCM) and cultured anaerobically at 37 °C for 5 days to obtain the first generation. Subsequently, 200 μL of this culture was transferred into fresh RCM and incubated under the same conditions for another 5 days to produce the second generation. Bacterial concentration was determined on Columbia blood agar plates and adjusted to ~3 × 10^8^ CFU/mL.

### Preparation and characterization of O-EVs

*O. splanchnicus* was cultured under appropriate anaerobic conditions until reaching a concentration of approximately 3 × 10^8^ CFU/mL. Extracellular vesicles were isolated from *O. supernatants* by sequential centrifugation (500 × g, 10 min; 2,000 × g, 20 min; 10,000 × g, 30 min) and 0.22 μm filtration. O-EVs were pelleted by ultracentrifugation (120,000 × g, 80 min, 4 °C), washed with PBS, and re-pelleted. Particle size and concentration were measured using a Zetasizer Nano S90, and morphology was examined by TEM on a JEM-1400 microscope at 100 kV after negative staining on 300-mesh grids.

### Lipidomics analysis

Lipidomic profiling was performed using a Q Exactive HFX high-resolution mass spectrometry platform. Samples were subjected to standardized pretreatment procedures to remove proteins and interfering substances, followed by extraction of lipid species. The resulting lipid extracts were analyzed by liquid chromatography–mass spectrometry (LC–MS) in both positive and negative ionization modes to acquire MS and MS/MS spectra. Lipid species were annotated and quantified using LipidSearch software, which was also applied for data preprocessing. A comprehensive lipid species list and the corresponding data matrix were generated for downstream analysis.

### H&E staining and histological assessment

Mouse lung tissues (~0.5 cm) were fixed in 4% paraformaldehyde, paraffin-embedded, sectioned (5–8 μm), dewaxed, rehydrated, and stained with H&E. Images were captured using a digital slide scanner (KF-PRO-002, KFBIO). Lung injury was scored semi-quantitatively (0–4) for edema, inflammation, hemorrhage, atelectasis, and hyaline membrane formation, with total scores calculated from 10 high-power fields per animal.

### Enzyme-linked immunosorbent assay

The concentrations of IL-1β, IL-6, and TNF-α in mouse serum were determined using IL-1β (MM-0040 M2, MEIMIAN, Jiangsu), TNF-α (MM-0132 M2, MEIMIAN, Jiangsu) and IL-6 (MM-0163 M2, MEIMIAN, Jiangsu) ELISA kits. Absorbance was measured at 450 nm using an Epoch microplate reader manufactured by BioTek (United States), thereby enabling the calculation of concentrations for each inflammatory cytokine.

### RNA isolation and quantitative Rt-PCR analysis

Mouse lung tissues were homogenized, and total RNA was extracted using TRIzol reagent. cDNA was synthesized using a reverse transcription kit, and quantitative PCR was performed with SYBR Green Master Mix on a LightCycler^®^96 system. Gene expression levels were normalized to β-actin and calculated using the 2^−ΔΔCt^ method. Primer sequences are listed in Supplementary File S1.

### 16S rRNA sequencing and gut microbiota analysis

Full-length 16S rRNA genes from intestinal contents were amplified and sequenced on the PacBio platform. Raw data were processed using SMRT Link (v5.0) to generate CCS reads, which were quality-filtered, denoised, and taxonomically annotated to determine microbial composition and relative abundance. Downstream analyses included α- and β-diversity, differential taxa identification, correlation analysis, and functional prediction.

### Macrophage phenotype analysis by flow cytometry

Mice were anesthetized, and tracheotomy followed by intubation was performed to collect bronchoalveolar lavage fluid (BALF). Lungs were lavaged three times with 1 mL of sterile PBS (total volume 2–2.5 mL). After filtration through a 70 μm cell filter, the collected BALF was resuspended in PBS to a final concentration of 1×10^6^ cells/mL. For flow cytometry, the macrophage population was identified by using reverse gating strategy. M1 macrophages were labeled with PE-conjugated anti-mouse CD68 (BioLegend, Cat: 137013), and M2 macrophages were labeled with Alexa Fluor 647-conjugated anti-mouse CD206 (BioLegend, Cat: 141711). By plotting CD68 and CD206, the double-positive population was identified, and their positions are traced back on the FSC/SSC scatter plot to optimize the gating strategy. Corresponding isotype controls were PE Rat IgG2a, κ (BioLegend, Cat: 400507) and Alexa Fluor 647 Rat IgG2a, κ (BD, Cat: 557690). Cells were fixed and permeabilized using Fixation Buffer and Permeabilisation Buffer (Biosharp, Cat: BL1146A).

### Western blotting

Total protein from mouse lung tissues was extracted using RIPA buffer, and concentrations were determined by BCA assay. Equal amounts of protein (50 μg) were separated by SDS–PAGE and transferred to PVDF membranes. After blocking with 5% skim milk, membranes were incubated overnight at 4 °C with primary antibodies against Cav1, Ces1d, and GAPDH, followed by incubation with secondary antibodies. Protein bands were visualized using a Tanon 5200SF imaging system and quantified by ImageJ. Target protein levels were normalized to GAPDH.

### RNA sequencing and analysis

The mRNA was enriched from total lung RNA using Oligo (dT) magnetic beads and fragmented for cDNA synthesis. First- and second-strand cDNA were generated using random hexamer primers, followed by end repair, A-tailing, adapter ligation, size selection, amplification, and purification. Library quality was assessed by Qubit, qPCR, and bioanalyzer. Qualified libraries were pooled and sequenced on an Illumina platform. Differential gene expression and functional enrichment analyses were subsequently performed.

### Co-immunoprecipitation

Protein A/G magnetic beads with the primary antibody (IgG or Cav1) were placed in a shaking incubator and incubated overnight at 4 °C. Mouse lung tissues were lysed using the same RIPA-based method described for Western blot analysis, and lysates were centrifuged at 3,000 × g for 10 min at 4 °C to collect the supernatant. Tissue lysates were then incubated with antibody-conjugated magnetic beads overnight at 4 °C. The captured proteins were eluted and subjected to mass spectrometry for identification of potential Cav1 interacting partners. Selected interactions were further validated by co-immunoprecipitation followed by Western blot analysis.

### Statistical analysis

Statistical analyses were performed using GraphPad Prism 9.5.1, and results are expressed as mean ± standard error (SEM). One way analysis of variance (ANOVA) was used for normally distributed and homogeneous data. For data that deviated from normality or exhibited unequal variance, k-independent-sample nonparametric tests were employed. Statistical significance was defined as *p* < 0.05.

## Results

### ALI induced by LPS was accompanied by severe pulmonary inflammation and gut microbiota dysbiosis

**Figure 1:**
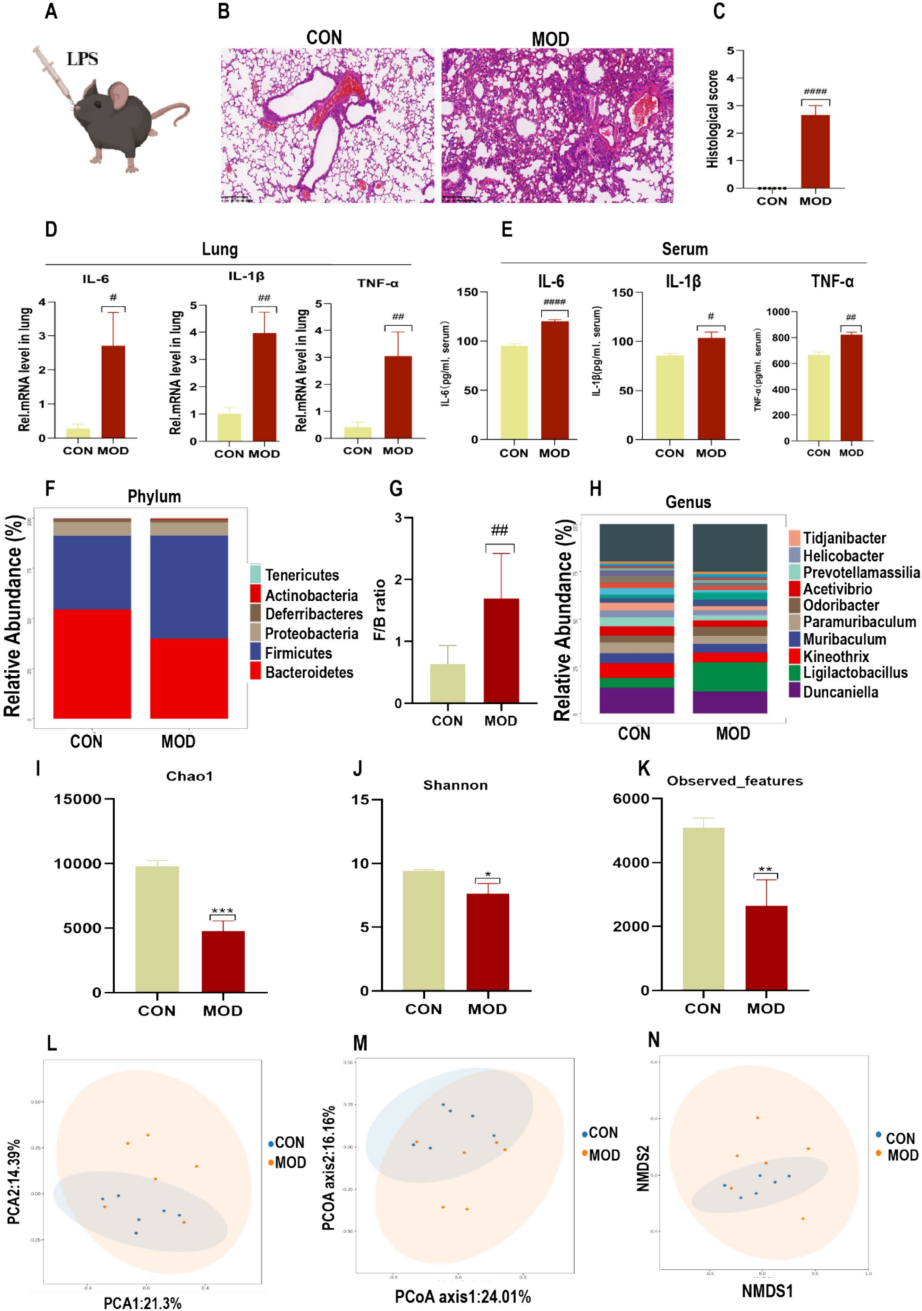
LPS administration induced acute lung injury and disrupted gut microbiota composition in mice. **(A)** Flowchart of animal procedures for the control group and model group. The modeling process utilizes LPS nasal inhalation. **(B)** H&E-stained histopathological section of lung tissue (Scale bar, 200 μm). **(C)** Quantitative histopathological scores of lung injury. **(D)** mRNA expression of pro-inflammatory cytokines (TNF-α, IL-1β, IL-6) in lung tissue. **(E)** Serum levels of TNF-α, IL-1β, and IL-6. **(F)** Relative abundance of dominant bacterial phylum. **(G)** Ratio of *Firmicutes* to *Bacteroidetes*. **(H)** Species richness of the intestinal flora at the genus level. **(I–K)** α-diversity analysis (Chao1, Shannon, Observed features). **(L)** Principal component analysis (PCA). **(M)** Principal coordinate analysis (PCoA). **(N)** Non-metric multidimensional scaling (NMDS1). Data are presented as mean ± SEM (*n* = 6). Statistical significance was assessed using one-way ANOVA followed by Tukey’s *post hoc* test. ^#^*p* < 0.05, ^##^*p* < 0.01, ^###^*p* < 0.001 vs. CON group; ^*^p < 0.05, ^**^p < 0.01, ^***^p < 0.001 vs. MOD group.

Based on previous literature research, certain specific intestinal bacteria was found to suppress the generation of inflammatory cytokines, thereby improving the survival rate of mice with ALI (Luo et al., 2021). This indicates that gut bacteria have potential for treating lung diseases. Therefore, we used LPS intranasal administration to induce an acute lung injury model in normal mice and observed whether there were changes in pathological indicators in mice with acute lung injury compared to normal mice (Figure 1A).

Histopathological examination revealed that MOD group induced severe pulmonary injury, characterized by extensive alveolar wall thickening, inflammatory cell infiltration, and interstitial congestion compared with the normal CON group (Figures 1B,C). To further characterize the inflammatory status, we examined cytokine expression in lung tissue and serum. The mRNA levels of IL-1β, IL-6, and TNF-α were significantly raised in MOD group, accompanied by corresponding increases in serum cytokine concentrations (Figures 1D,E). These results demonstrate that LPS-induced ALI triggered a pronounced pulmonary and systemic inflammatory response.

Given the involvement of the gut–lung axis in pulmonary inflammation, we analyzed gut microbiota alterations. At the phylum level, LPS exposure decreased *Bacteroidetes* and increased *Firmicutes*, resulting in a significantly elevated *Firmicutes/Bacteroidetes*, (F/B) ratio (Figures 1F,G), indicative of microbial dysbiosis. At the genus level, beneficial taxa including *Muribaculaceae, Lactobacillus,* and *Bacteroides* were markedly reduced, whereas pro-inflammatory genera such as *Romboutsia* and *Turicibacter* were enriched in the ALI group (Figure 1H). Consistently, α-diversity indices (Chao1, Shannon, and Simpson) were significantly decreased, reflecting reduced microbial richness and evenness (Figures 1I–K). β-diversity analysis further demonstrated clear separation between the CON and MOD groups in PCoA plots (Figures 1L–N), indicating pronounced gut microbiota remodeling following LPS challenge.

### FMT confirmed that the development of ALI is associated with the gut microbiota

**Figure 2:**
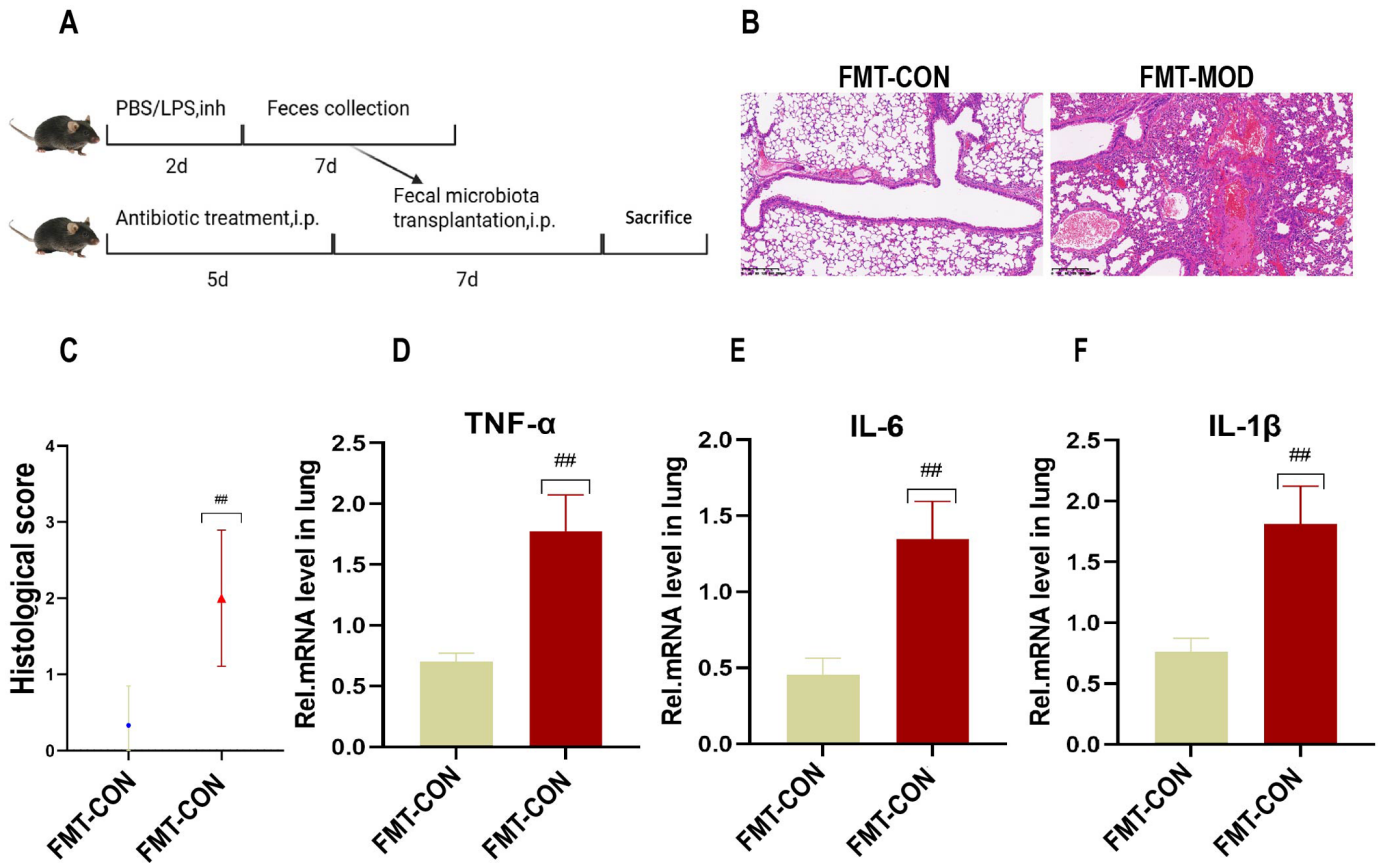
FMT reveals that gut microbial dysbiosis contributes to LPS-induced acute lung injury. **(A)** FMT experimental procedure. **(B)** H&E-stained histopathological section of lung tissue (Scale bar, 200 μm). **(C)** Quantitative histopathological scores of lung injury. **(D–F)** mRNA expression of pro-inflammatory cytokines (TNF-α, IL-1β, IL-6) in lung tissue. Data are presented as mean ± SEM (*n* = 6). Statistical significance was assessed using one-way ANOVA followed by Tukey’s *post hoc* test. ^##^*p* < 0.01, vs. CON group.

Given the observed dysbiosis in LPS-induced ALI mice, FMT was performed to determine whether dysbiosis of the gut microbiota contribute to pulmonary inflammation (Figure 2A). Pseudo-germ-free mice colonized with fecal microbiota from ALI donors exhibited aggravated lung injury compared with those receiving microbiota from healthy controls. Histopathological examination revealed pronounced alveolar wall thickening, inflammatory cell infiltration, and hemorrhage in the lungs of the ALI microbiota–transplanted group (Figures 2B,C).

Consistently, mRNA levels of TNF-α, IL-1β, and IL-6 were markedly elevated in lung tissues of FMT-MOD group relative to FMT-CON group (Figures 2D–F). Our findings demonstrate that the gut microbiota from ALI mice is a crucial factor in exacerbating pulmonary inflammation and tissue injury, highlighting the pivotal effects of intestinal dysbiosis on mediating the gut–lung axis during acute lung injury.

### *Odoribacter splanchnicus* is a decisive bacterium in the development of ALI

**Figure 3:**
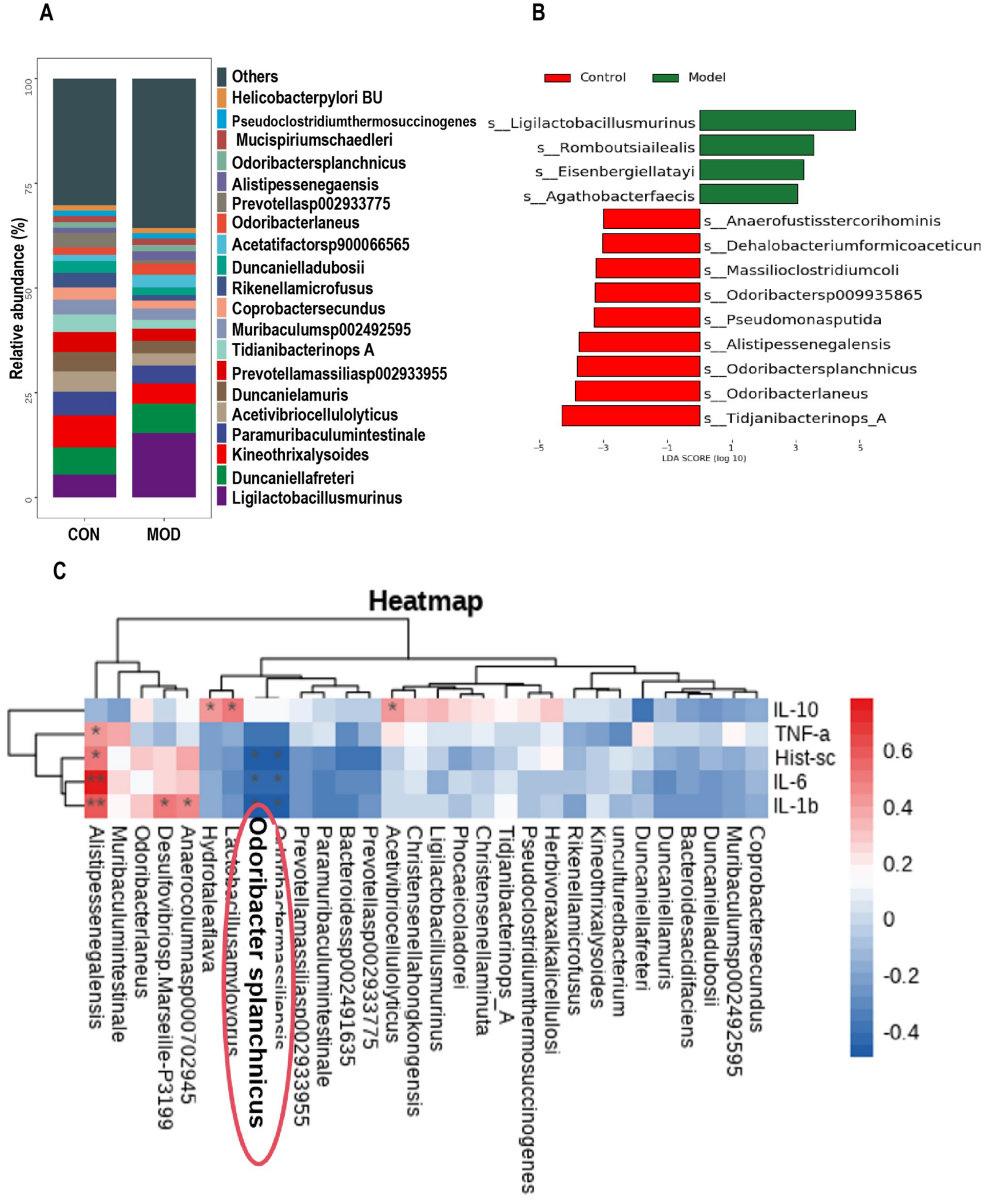
Differential abundance of beneficial bacteria and their association with inflammatory responses in acute lung injury (ALI). **(A)** Analysis of relative abundance at the species level of gut microbiota in the CON and MOD groups. **(B)** Lefse analysis. **(C)** Spearman’s correlation analysis between the top 30 differential bacterial species and inflammatory markers.

Given the evidence that gut dysbiosis contributes to the etiopathogenesis of ALI, we further sought to identify specific bacterial taxa potentially driving this process. At the species level, the top 20 beneficial bacteria identified included *Duncanella freteri*, *Alistipes senegalensis*, and *Odoribacter splanchnicus*, among others (Figure 3A). To further assess the differential abundance of these key taxa, LefSe analysis was conducted. The results revealed that *Tidjanibacter inops A*, *Odoribacter laneus*, *Odoribacter splanchnicus*, *Alistipes senegalensis*, *Pseudomonas putida*, *Odoribacter sp009935865*, *Massilioclostridium coli*, *Dehalobacterium formicoaceticum*, and *Anaerofustis stercorihominis* showed a substantial decrease in abundance compared to the CON group. In contrast, *Ligilactobacillus murinus*, *Romboutsia ilealis*, *Eisenbergiella tayi*, and *Agathobacter faecis* exhibited a significant increase in abundance (Figure 3B). To further elucidate this relationship, Spearman’s correlation analysis was conducted between major bacterial species and inflammatory indices. *O. splanchnicus* abundance exhibited a strong negative correlation with pro-inflammatory cytokines IL-1β, IL-6, and TNF-α, also with histopathological injury scores (Figure 3C). Taken together, these integrated analyses progressively identified *O. splanchnicus* as a key bacterial species closely associated with inflammatory regulation and tissue injury in ALI, suggesting that its depletion may contribute to gut–lung axis dysfunction during disease progression.

### *Odoribacter splanchnicus* significantly alleviates ALI induced by LPS

**Figure 4:**
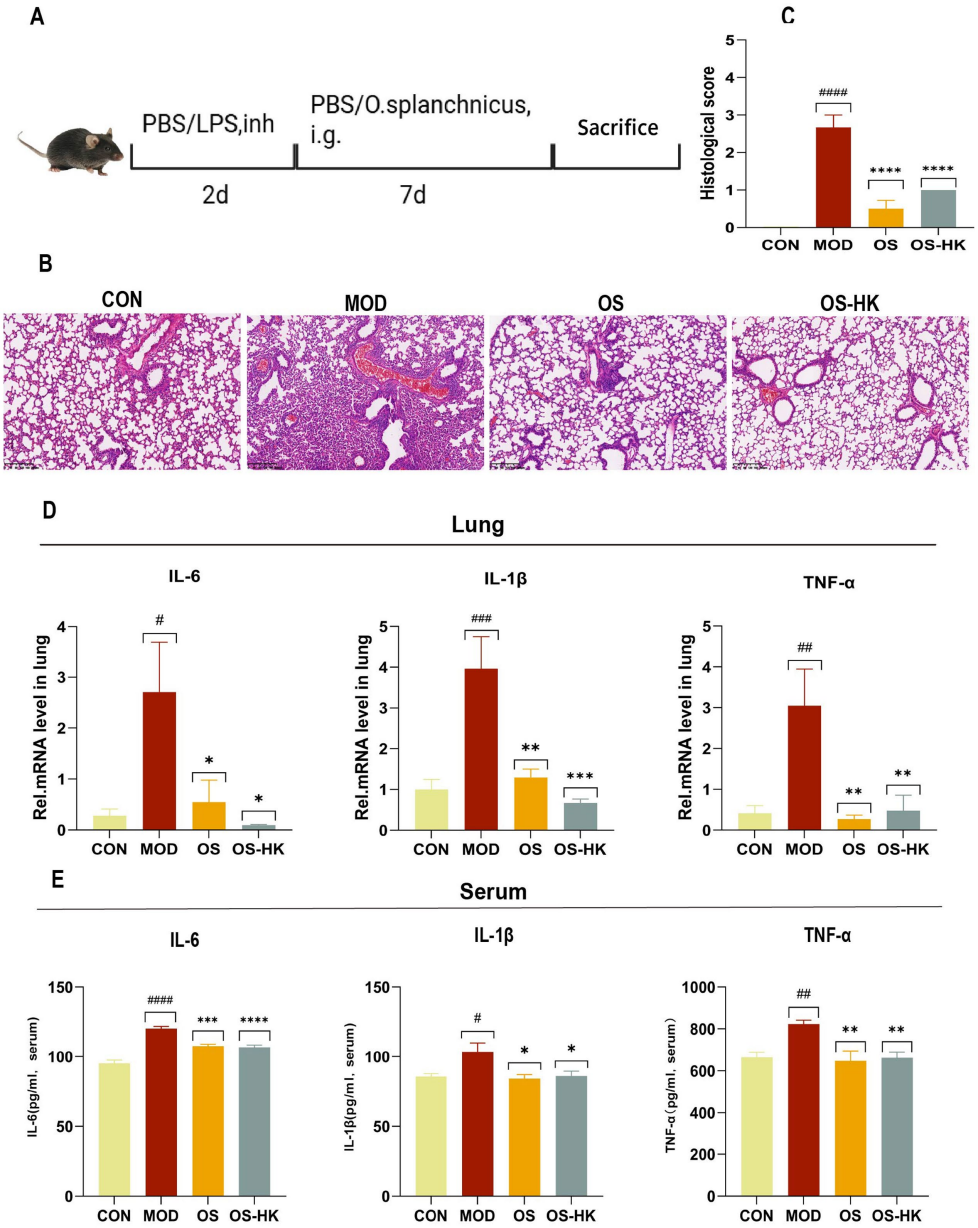
Therapeutic effects of *O. splanchnicus* on ALI in mice. **(A)** Pharmacodynamic experimental procedure of *O. splanchnicus* in ALI models. **(B,C)** H&E-stained histopathological lung tissue sections from CON, MOD, OS, and OS-HK groups. **(D)** Transcriptional expression levels of pro-inflammatory cytokines TNF-α, IL-1β, and IL-6 in lung tissue. **(E)** Serum levels of pro-inflammatory cytokines TNF-α, IL-1β, and IL-6. Data are presented as mean ± SEM (*n* = 6). Statistical significance was assessed using one-way ANOVA followed by Tukey’s post hoc test. ^#^*p* < 0.05, ^##^*p* < 0.01, ^###^*p* < 0.001 vs. CON group; ^*^p < 0.05, ^**^p < 0.01, ^***^p < 0.001 vs. MOD group.

To directly assess the impact of *O. splanchnicus* on ALI, a pharmacodynamic experiment was conducted using both live (OS) and heat-killed *O. splanchnicus* (OS-HK) in ALI mice (Figure 4A). In the MOD group, severe inflammation was observed, with thickened alveolar walls and significant inflammatory cell infiltration (Figure 4B). In contrast, both treatment groups showed reduced alveolar damage, alleviated inflammatory infiltration, and less thickening of the alveolar walls (Figures 4B,C). Moreover, the expression levels of IL-1β, TNF-α, and IL-6 were significantly reduced in both serum and lung tissue from the OS and OS-HK groups compared with the MOD group (Figures 4D,E). Notably, both treatments exhibited similar therapeutic effects, suggesting that the beneficial effects of *O. splanchnicus* do not require its viability.

### Isolation and lipidomic features of *Odoribacter splanchnicus*-derived outer membrane vesicles

Given that both live and heat-killed *O. splanchnicus* strains alleviated ALI in mice, we hypothesized that bacterial extracellular vesicles derived from *O. splanchnicus* (O-EVs) might mediate these protective effects. Since EVs serve as pivotal agents of intercellular communication, carrying and delivering bacterial bioactive molecules independent of live bacterial metabolism, their role in ALI warranted investigation.

**Figure 5:**
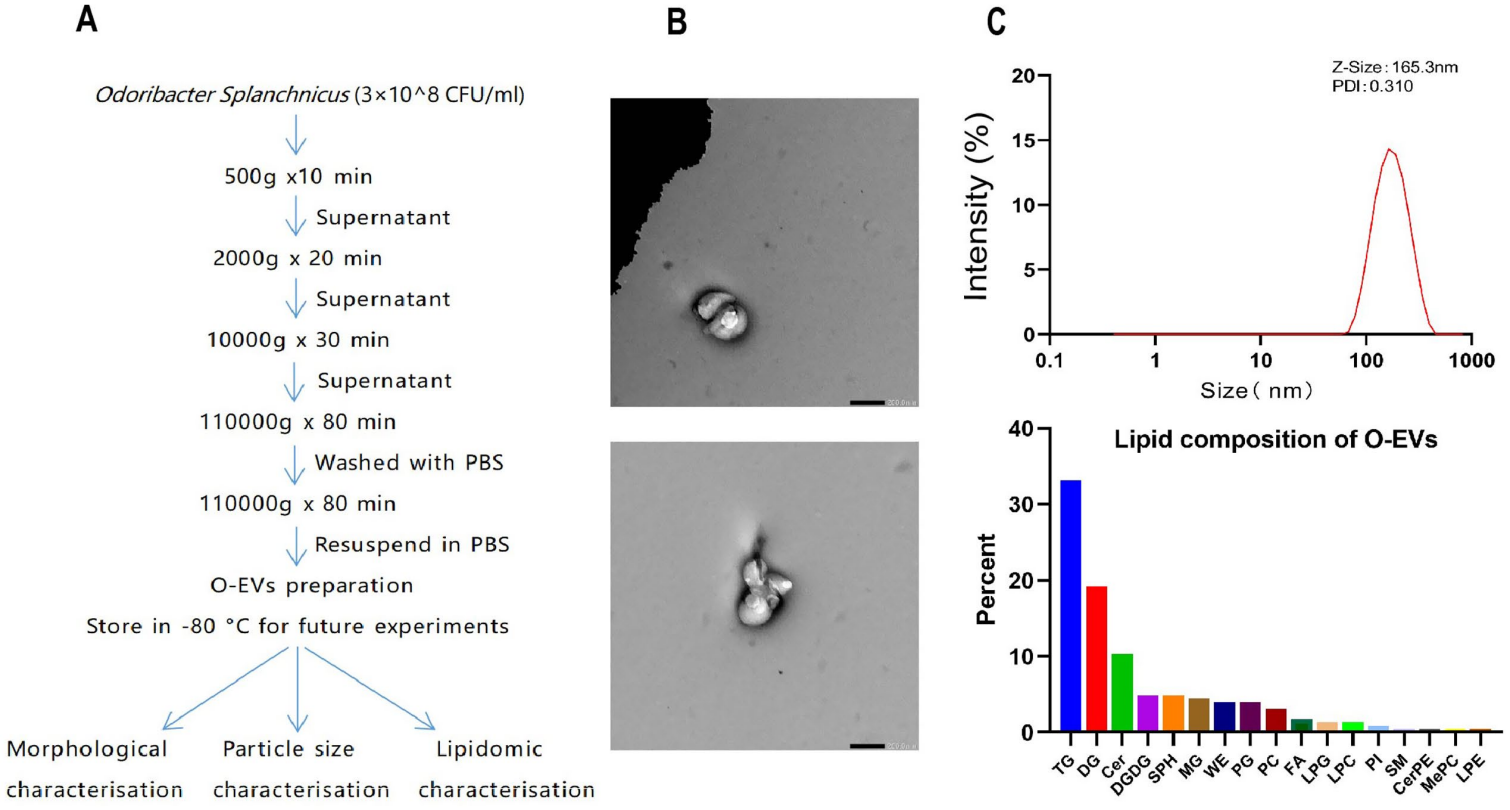
Isolation and lipidomic features of O-EVS. **(A)** Extraction and purification of O-EVs by differential ultracentrifugation. **(B)** Morphological characterization of O-EVs under transmission electron microscopy. **(C)** Particle size distribution of purified O-EVs. **(D)** Lipidomic features of O-EVS.

O-EVs were isolated from bacterial culture supernatants through sequential centrifugation and ultracentrifugation (Figure 5A). The obtained O-EVs were resuspended in PBS to formulate the O-EVs preparation. Transmission electron microscopy indicated that O-EVs preparation exhibit a characteristic cup-shaped vesicular structures with intact membranes (Figure 5B). Dynamic light scattering analysis confirmed a homogeneous size distribution with a mean diameter of 165.3 nm and a polydispersity index of 0.31 (Figure 5C), indicating successful purification of O-EVs.

Given that the relative composition of membrane lipids is closely associated with metabolic and inflammatory states, lipidomic analysis was performed to characterize O-EVs preparation (Figure 5D). Neutral lipids predominated the lipidome, with triacylglycerols (TG) and diacylglycerols (DG) accounting for 33.2 and 19.3% of total lipids, respectively, together comprising more than 50%. These lipid classes are commonly associated with enhanced membrane flexibility and curvature modulation, and their high abundance reflects increased structural plasticity and stability of the vesicle membrane (Ghadami and Dellinger, 2023).

The lipidomic profile of O-EVs revealed enrichment of bacterial-associated sphingolipids, including multiple ceramide species, sphingosine bases with d17–d18 backbones, and ceramide phosphoethanolamine, together with anionic phospholipids such as phosphatidylglycerol and phosphatidylinositol (Supplementary File S2). These lipid species have been reported to participate in immune homeostasis by modulating innate immune signaling and macrophage inflammatory responses (Uhlig and Yang, 2013; Petrusca et al., 2010). In particular, bacterial-derived sphingolipids differ functionally from host stress-associated ceramides and have been shown to constrain excessive inflammatory activation (Uhlig and Yang, 2013). Meanwhile, phosphatidylglycerol is a recognized anti-inflammatory component in pulmonary tissues, capable of suppressing Toll-like receptor–mediated signaling, whereas phosphatidylinositol provides a signaling scaffold for PI3K-dependent pathways involved in macrophage functional regulation (Kuronuma et al., 2009). Collectively, the lipidomic features of O-EVs indicate that, in addition to providing structural integrity, these vesicles contain lipid species with potential immunological relevance, suggesting a possible association between O-EVs and anti-inflammatory effects in the context of acute lung injury.

### O-EVs play a key protective role against ALI

**Figure 6:**
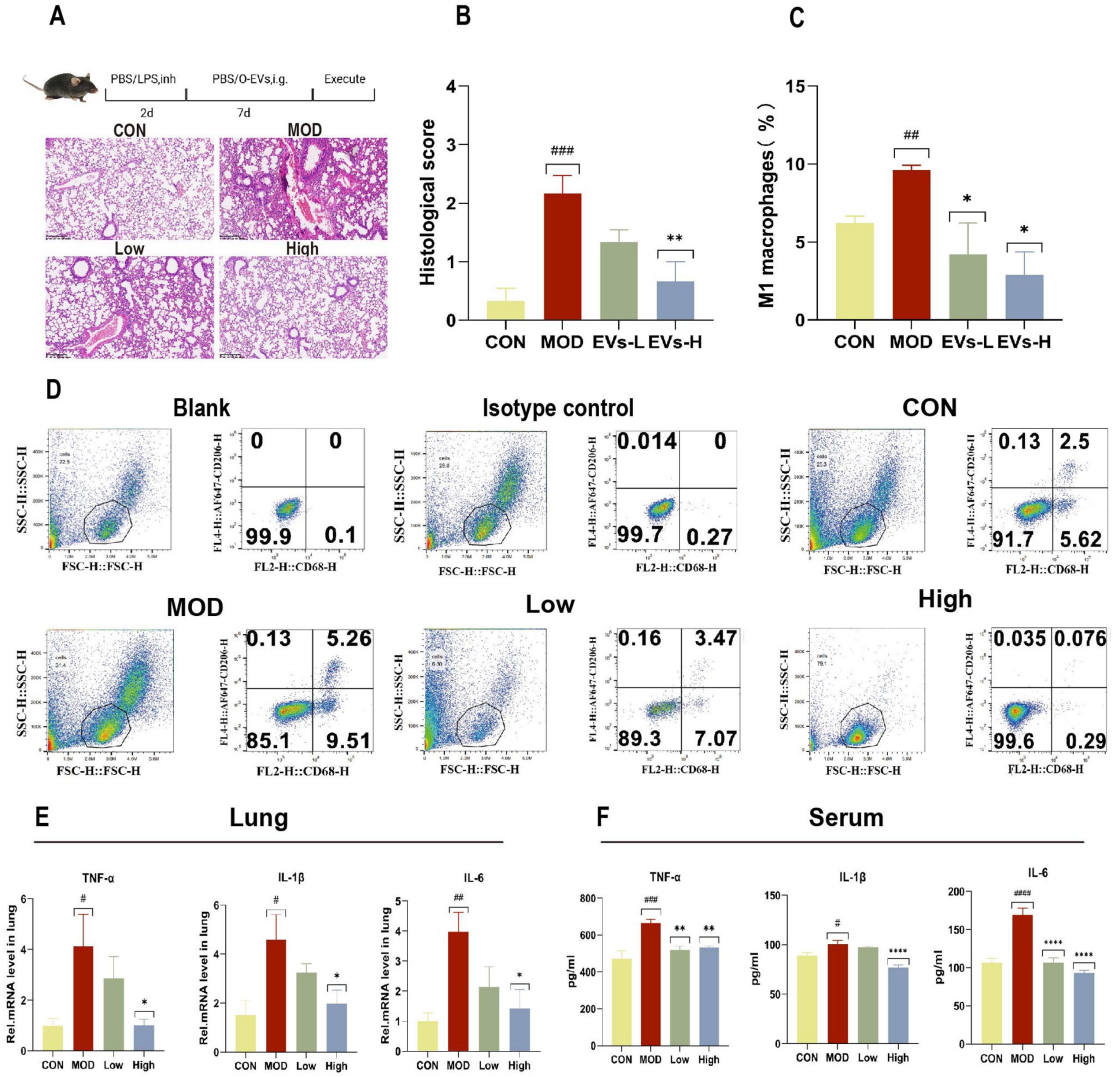
O-EV*s* are major mediators of protection against ALI. **(A)** Schematic of animal experiments evaluating the pharmacodynamic effects of O-EVs. **(B)** Representative H&E-stained lung sections from CON, MOD, EVs-L, and EVs-H groups. **(C)** Percentage of M1 macrophages in bronchoalveolar lavage fluid. **(D)** Flow cytometry analysis of alveolar macrophage phenotypes in each group (M1: CD68^+^CD206^−^; M2: CD68^−^CD206^+^). **(E)** mRNA expression of proinflammatory cytokines (TNF-α, IL-1β, IL-6) in lung tissue. **(F)** Serum levels of proinflammatory cytokines (TNF-α, IL-1β, IL-6). Data are presented as mean ± SEM (*n* = 6). Statistical significance was assessed using one-way ANOVA followed by Tukey’s *post hoc* test. ^#^*p* < 0.05, ^##^*p* < 0.01, ^###^*p* < 0.001, ^####^*p* < 0.0001 vs. CON group; ^*^p < 0.05, ^**^p < 0.01, ^***^p < 0.001, ^****^p < 0.0001 vs. MOD group.

To evaluate O-EVs pharmacological activity, ALI mice induced by LPS were treated with O-EVs via intragastric administration (Figure 6A). Histopathological examination showed that O-EV treatment markedly ameliorated alveolar wall thickening, inflammatory cell infiltration, and pulmonary edema compared with the model (MOD) group (Figure 6B). Quantitative scoring confirmed a significant reduction in histological injury, particularly in the high-dose O-EV group (Figure 6C). Flow cytometric analysis of alveolar macrophages identified elevated CD68^+^/CD86^+^ M1-type populations in the MOD group, which were markedly reduced in both EVs-L and EVs-H groups, without significant change in CD206^+^ M2 macrophages (Figure 6D). Correspondingly, mRNA levels of IL-1β, TNF-α, and IL-6 in lung tissues were significantly downregulated after O-EVs treatment (Figure 6E). Consistent with this, serum levels of these proinflammatory cytokines were significantly reduced, confirming the systemic anti-inflammatory effect of O-EVs (Figure 6F).

### O-EVs alleviates ALI by modulating the interaction between Cav1 and Ces1d

**Figure 7:**
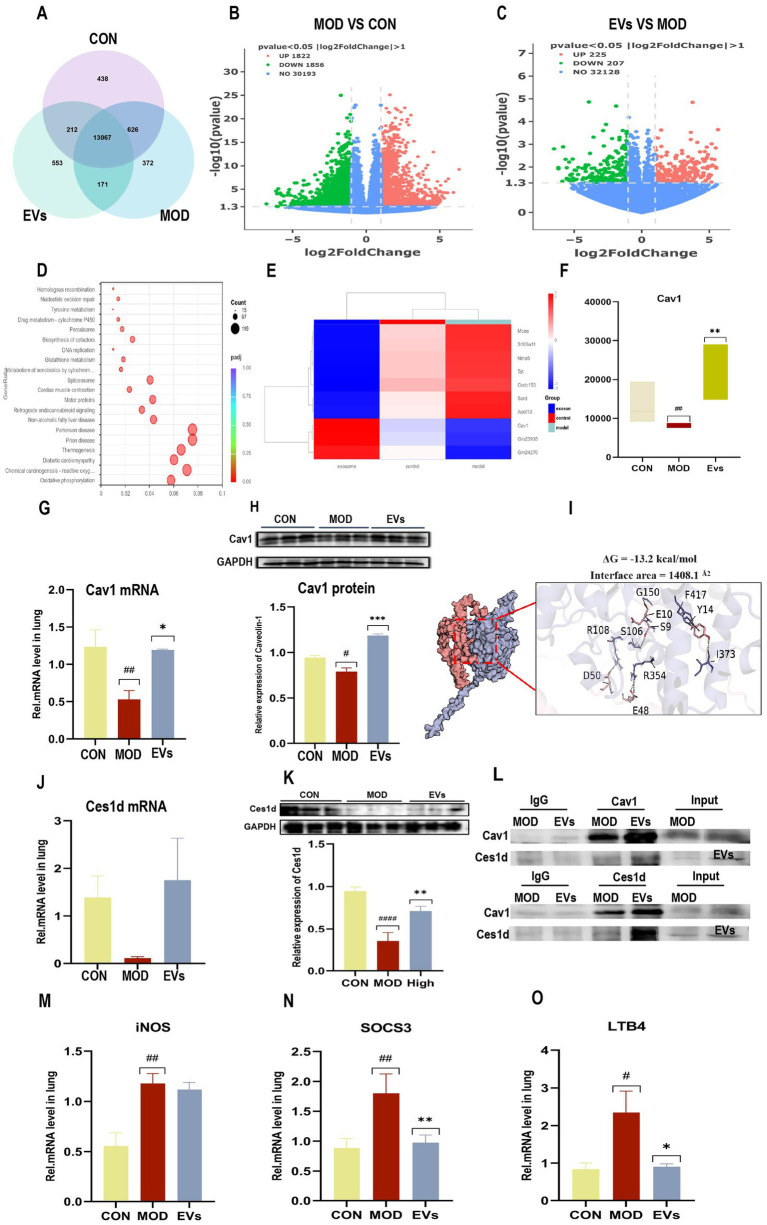
Transcriptomic and Co-IP analysis reveals O-EVs enhanced Cav1-Ces1d interaction in the hippocampus. **(A)** Venn diagram. **(B)** Volcano plot (MOD VS CON). **(C)** Volcano plot (EVs VS MOD). **(D)** KEGG pathway. **(E)** Clustering analysis of differentially expressed genes in transcriptomics. **(F)** Expression of Cav1 in different groups in transcriptomics. **(G,H,J,K)** RT-qPCR and Western blot validation of Cav1 and Ces1d expression in lung tissues. **(I)** Protein–protein interaction prediction. **(L)** Co-ip and Western blot analysis validated the protein interaction between Cav1 and Ces1d. **(M–O)** mRNA expression of proinflammatory cytokines (iNOS, SOCS3, LTB4) in lung tissue. Data are presented as mean ± SEM (*n* = 6). Statistical significance was assessed using one-way ANOVA followed by Tukey’s *post hoc* test. ^#^*p* < 0.05, ^##^*p* < 0.01, ^###^*p* < 0.001, ^####^*p* < 0.0001 vs. CON group; ^*^p < 0.05, ^**^p < 0.01, ^***^p < 0.001 vs. MOD group.

To further elucidate the molecular mechanisms by which *O. splanchnicus*–derived extracellular vesicles (O-EVs) mitigate acute lung injury (ALI), we performed transcriptomic profiling of lung tissues from the CON, MOD, and EVs groups. A total of 13,067 genes were commonly expressed across all three groups (Figure 7A). Differential gene expression analysis revealed that, compared with the control group, the MOD group exhibited 1822 genes upregulated and 1856 genes downregulated genes (Figure 7B). Relative to the MOD group, theEVs-treated group showed 225 genes upregulated and 207 genes downregulated (Figure 7C). KEGG pathway enrichment revealed a significant upregulation of protective metabolic pathways following O - EVs treatment, including oxidative phosphorylation, glutathione metabolism, peroxisome, and drug metabolism – cytochrome P450, which are closely associated with mitochondrial energy production and antioxidant defense (Figure 7D). Through transcriptomic differential gene clustering analysis, we identified several significantly altered genes following O-EVs treatment. Among them, Cav1, Gm23935, and Gm24270 were markedly upregulated compared with the MOD group. Notably, Cav1 is a key structural component of caveolae involved in maintaining endothelial integrity and regulating inflammatory signaling (Figures 7E,F). To validate the transcriptomic findings, we performed qPCR and Western blot analyses, both confirming the restoration of Cav1 expression after O-EVs treatment (Figures 7G,H).

Building upon these results, we further explored the potential downstream interactions of Cav1. Co-IP coupled with mass spectrometry identified Ces1d as a putative Cav1-interacting protein (Supplementary File S3). To validate Ces1d as an interacting protein of CAV-1, we employed Alphafold 3 to predict protein–protein interactions between Cav-1 and Ces1d. The predicted results were visualized using PyMOL 3.0.3, revealing a binding energy of −13.2 kcal/mol between two proteins and a contact area of 1408.1 Å^2^ (Figure 7I). Subsequent qPCR and Western blot analyses revealed a parallel expression pattern between Ces1d and Cav1 across all groups (Figures 7J,K), implying a functional association between these molecules. To substantiate this hypothesis, we performed Co-IP and Western blot analyses to examine Cav1-Ces1d co-expression (Figure 7L). Consistent with our speculation, the Cav1-Ces1d interaction was markedly weakened in the MOD group but significantly enhanced following O-EVs administration. These findings collectively suggest that O-EVs may mitigate acute lung injury by reinforcing the Cav1-Ces1d interaction, thereby restoring alveolar barrier function and attenuating pulmonary inflammation.

Cav1 is a key negative regulator of NF-κB- and STAT3-dependent inflammatory signaling (Fan et al., 2024), whereas Ces1d is critically involved in the regulation of lipid-derived inflammatory mediators in lung tissue (Szafran et al., 2022). Given the enhanced Cav1–Ces1d interaction induced by O-EVs, we next examined whether O-EVs modulate the downstream signaling pathways specifically associated with Cav1 and Ces1d. In the LPS-induced acute pneumonia model, inflammatory signaling downstream of Cav1 was markedly activated, as evidenced by significantly elevated pulmonary expression of TNF-α, IL-1β, and IL-6 (Figure 6E), together with increased levels of iNOS and SOCS3 (Figures 7M,N), two representative outputs of NF-κB and STAT3 signaling. O-EVs administration significantly reduced the expression of these cytokines and inflammatory mediators, indicating effective suppression of Cav1-dependent inflammatory signaling. Concurrently, lipid inflammatory pathways associated with Ces1d were also profoundly dysregulated in the model group. The level of leukotriene B4 (LTB4), a potent lipid chemoattractant generated through Ces1d-associated lipid metabolic processes, was markedly increased (Figure 7O). Treatment with O-EVs significantly decreased pulmonary LTB4 levels, indicating attenuation of Ces1d-related lipid inflammatory mediator production. Importantly, the inhibition of Cav1-dependent inflammatory signaling and the reduction of Ces1d-associated lipid mediator generation occurred in parallel with restoration of Cav1 expression and reinforcement of the Cav1–Ces1d interaction. These coordinated changes suggest that O-EVs do not act on Cav1 or Ces1d in isolation but rather promote a functional coupling between inflammatory signaling control and lipid metabolic regulation. Through this coordinated modulation of Cav1- and Ces1d-dependent downstream pathways, O-EVs effectively limit inflammatory amplification during acute lung injury.

## Discussion and conclusion

The pathophysiology of ALI involves uncontrolled inflammation, disruption of epithelial and endothelial barriers, and dysregulated immune responses (Ramji et al., 2023). Recent evidence underscores the importance of the gut–lung axis in modulating pulmonary immunity and inflammation. In this study, we demonstrate that *O. splanchnicus*, a commensal gut bacterium, and its derived extracellular vesicles ameliorate LPS-induced ALI in mice by enhancing the Cav1–Ces1d interaction. This regulatory axis is associated with coordinated modulation of NF-κB- and STAT3-related inflammatory signaling and suppression of lipid-derived inflammatory mediators, thereby linking microbial vesicle signaling to inflammatory control in distant organs. Our findings reveal a novel mechanism whereby gut microbiota-derived vesicles influence damage to distant organs, while simultaneously highlighting their therapeutic potential.

We first established that LPS-induced ALI leads to significant gut microbiota dysbiosis, characterized by a reduction in *O. splanchnicus* abundance. This finding aligns with recent studies indicating that systemic inflammation and lung injury can alter gut microbial composition, further exacerbating pulmonary pathology through immune cross-talk (Ren et al., 2024). The observed decrease in *O. splanchnicus* is particularly relevant given its known anti-inflammatory properties and association with metabolic health (Lima et al., 2022). Subsequent FMT experiments confirmed the functional effect of the gut microbiota in ALI. Mice receiving fecal transplants from ALI donors exhibited worsened lung injury and heightened inflammation, whereas transplantation from *O. splanchnicus*-supplemented donors mitigated these effects. These results emphasize the causal role of gut microbial composition in mediating lung inflammation and injury, consistent with the concept of gut–lung axis (Yoon et al., 2022).

Notably, both live and inactivated *O. splanchnicus* were effective in attenuating ALI, suggesting that the protective effects are likely mediated by bacterial components rather than viable metabolic activity. This led us to investigate bacterial extracellular vesicles as key mediators. Bacterial extracellular vesicles have emerged as important vehicles for interkingdom communication, capable of transporting bioactive molecules across biological barriers and influencing host immunity (Diaz-Garrido et al., 2021). Our isolation and characterization of O-EVs revealed nanoparticles approximately 165 nm in diameter with typical cup-shaped morphology, consistent with previously described bacterial extracellular vesicles (Aytar et al., 2022). Administration of O-EVs significantly reduced lung histopathology scores, inflammatory cytokine levels, and alveolar macrophage M1 polarization. These effects were dose-dependent, with higher doses of O-EVs yielding greater protection. The conversion in macrophage polarization from pro-inflammatory M1 phenotype to anti-inflammatory M2 phenotype is a critical mechanism in resolving ALI, as M1 macrophages are known to drive neutrophilic infiltration and tissue damage (He et al., 2023). Our flow cytometry data clearly demonstrate that O-EVs reduce the proportion of M1 macrophages without significantly altering M2 populations, suggesting a targeted immunomodulatory action.

To elucidate the molecular mechanisms underlying O-EV-mediated protection, we focused on Cav1 and Ces1d, both of which are critically involved in endothelial barrier integrity and inflammatory regulation. Cav1 is a key structural protein of caveolae that maintains vascular homeostasis and negatively regulates inflammatory signaling pathways, including NF-κB and STAT3, thereby limiting inflammatory cell infiltration and cytokine production (Garrean et al., 2006; Zhang et al., 2022; Dalton et al., 2023). Consistently, reduced Cav1 expression has been associated with increased vascular permeability and exacerbated acute lung injury (ALI) (Maniatis et al., 2012). In parallel, Ces1d, a lipid-metabolizing esterase abundantly expressed in lung tissue, has recently emerged as an important regulator of inflammation and cellular stress responses, partly through modulation of lipid-derived inflammatory mediators (Shao et al., 2025). In this study, we found that O-EV treatment markedly attenuated the upregulation of pro-inflammatory cytokines (TNF-α, IL-1β, and IL-6), inflammatory mediators (iNOS and SOCS3), and the potent lipid chemoattractant leukotriene B4 (LTB4) in an acute pneumonia model. These molecules are well-established downstream effectors of NF-κB- and STAT3-dependent inflammatory cascades and play central roles in amplifying lung inflammation and leukocyte recruitment during ALI. The coordinated suppression of cytokine signaling and LTB4 production suggests that O-EVs exert a broad anti-inflammatory effect by simultaneously restraining inflammatory transcriptional programs and lipid mediator–driven immune activation.

Notably, our lipidomic analysis revealed that O-EVs are markedly enriched in bacteria-specific sphingolipids, and these overrepresented lipid classes are highly consistent with the functional characteristics of Cav1 and Ces1d. As a key structural protein of caveolae, Cav1 plays a central role in maintaining endothelial barrier integrity and also serves as an essential organizer of plasma membrane lipid microdomains (lipid rafts), directly contributing to the formation and signal integration of sphingolipid- and cholesterol-enriched membrane regions (Bhowmick et al., 2023). In parallel, Ces1d, a carboxylesterase 1d highly expressed in lung tissue, represents a critical regulator of lipid metabolism and modulates the availability of intracellular lipid signaling molecules through hydrolysis of lipid ester bonds, particularly influencing the metabolic fate of pro-inflammatory lipid precursors such as arachidonic acid (Shao et al., 2025). Based on these findings, we propose that O-EVs may deliver these bioactive lipids to the lung and thereby establish a dual “structural–metabolic” regulatory mechanism. On the one hand, enriched sphingolipids may incorporate into host cell membranes and alter the lipid composition and biophysical properties of lipid rafts, thereby promoting Cav1 clustering within membrane microdomains, enhancing its function as a signaling hub, and reinforcing endothelial barrier stability. On the other hand, these exogenous lipid substrates and their metabolic derivatives may act as regulatory cues for Ces1d, driving lipid metabolic reprogramming and suppressing the production of pro-inflammatory lipid mediators such as leukotriene B4. Furthermore, the functional interplay between Cav1 and Ces1d may constitute a key integrated node linking transmembrane signal transduction with intracellular lipid metabolism: Cav1 constrains inflammatory transcriptional programs by inhibiting NF-κB and STAT3 signaling, whereas Ces1d limits leukocyte activation and infiltration by restricting the generation of lipid inflammatory mediators. Collectively, these mechanisms may enable O-EVs to coordinately establish an effective anti-inflammatory defense at both the membrane signaling platform and lipid metabolic network levels.

The ability of O-EVs to modulate the Cav1/Ces1d axis highlights the therapeutic potential of bacterial extracellular vesicles as nanotherapeutics. Compared with live bacteria, bacterial extracellular vesicles are non-replicative, structurally stable, and amenable to engineering for targeted delivery (Munoz-Echeverri et al., 2024). To our knowledge, this study provides evidence for the therapeutic efficacy of O-EVs in ALI and suggests a mechanistic link between their protective effects and Cav1/Ces1d signaling. These findings are consistent with emerging evidence that bacterial extracellular vesicles derived from beneficial commensals, such as *Bacteroides thetaiotaomicron*, can elicit robust anti-inflammatory responses (Fonseca et al., 2023). Nevertheless, although we identified Cav1/Ces1d as a critical signaling node, the specific bioactive cargos within O-EVs—such as proteins, lipids, or nucleic acids—responsible for initiating this regulatory interaction remain to be defined and warrant further investigation.

In summary, our findings identify the gut commensal *O. splanchnicus* as an active regulator of gut–lung axis communication in lipopolysaccharide-induced acute lung injury, with its extracellular vesicles serving as key effectors of this distal protection. Through vesicle-mediated signaling, *O. splanchnicus* reinforces the functional interaction between Cav1 and Ces1d, thereby coordinately restraining NF-κB- and STAT3-dependent inflammatory signaling, limiting pro-inflammatory cytokine production, and suppressing lipid-derived inflammatory mediator generation. This bacterium-centered mechanism expands current understanding of how specific gut microbes, rather than the microbiota as a whole, orchestrate inflammatory control in distant organs via extracellular vesicles.

## Data Availability

The datasets presented in this study can be found in online repositories. The names of the repository/repositories and accession number(s) can be found in the article/Supplementary material.
