# Supplementary material for: Odoribacter splanchnicus elicits lung protection via vesicle-driven enhancement of the host Cav1–Ces1d interaction
Source: Front Microbiol. 2026 Jul 7;17:1860203. doi: 10.3389/fmicb.2026.1860203 (PMC13385493; doi:10.3389/fmicb.2026.1860203)
Supplement: Supplementary file 1 [file Table_1.DOCX]

Table 1.Primer sequences used in this study.

| **Gene name** | **Forward Sequence (5'-3')** | **Reverse Sequence (5'-3')** |
| --- | --- | --- |
| IL-1β | AAGAAAGAAAGTGGGTGGGCA | CCTATACAACGGCTCCTCCG |
| IL-6 | TTCAACCAAGAGGTGAGTGCT | GTCTGGAAAAAGTGCCGCTAC |
| TNF-α | ACTGATGAGAGGGAGGCCAT | CCGTGGGTTGGACAGATGAA |
| Cav-1 | GGGAACAGGGCAACATC | TGCTTCTCAGTCACCTCGT |
| Ces1d | TGCTCTCAGGATGCTGTTGG | AGGGCCAGTCCATCATAGGT |
| β-actin | GAAGGCTATAGTCACCTCGGG | ATGGTAATAATGCGGCCGGT |
