# Supplementary material for: Odoribacter splanchnicus elicits lung protection via vesicle-driven enhancement of the host Cav1–Ces1d interaction
Source: Front Microbiol. 2026 Jul 7;17:1860203. doi: 10.3389/fmicb.2026.1860203 (PMC13385493; doi:10.3389/fmicb.2026.1860203)
Supplement: Supplementary file 3 [file Table_3.DOCX]

**Table 1. LC–MS Detection Results of Proteins Co-immunoprecipitated with Cav1**

| gene | Protein Length | Protein Probability | Combined Total Peptides | Combined Unique Spectral Count | Combined Total Spectral Count | Cav1 Unique Spectral Count | Cav1 Total Spectral Count | Cav1 Intensity |
| --- | --- | --- | --- | --- | --- | --- | --- | --- |
| Ces1d | 565 | 1 | 8 | 7 | 9 | 7 | 9 | 189094.39 |
| Sf1 | 653 | 0.9999 | 3 | 3 | 3 | 2 | 2 | 26539.89 |
| Dync1i2 | 612 | 0.9998 | 2 | 2 | 2 | 2 | 2 | 66171.72 |
| Eif4a2 | 407 | 0.9998 | 11 | 1 | 36 | 1 | 21 | 37542.63 |
| Sars1 | 512 | 0.9998 | 2 | 2 | 2 | 1 | 1 | 22255.44 |
| Hpx | 460 | 0.9998 | 3 | 3 | 3 | 3 | 3 | 112453.55 |
| Adam17 | 827 | 0.9998 | 2 | 3 | 3 | 2 | 2 | 32740.088 |
| Krt13 | 437 | 0.9996 | 13 | 2 | 113 | 2 | 64 | 139552.55 |
| Alcam | 583 | 0.9996 | 2 | 2 | 2 | 2 | 2 | 73270.56 |
| Trim34b | 485 | 0.9988 | 2 | 0 | 2 | 0 | 1 | 105584.11 |
| Sypl1 | 261 | 0.9982 | 1 | 2 | 2 | 2 | 2 | 66419.24 |
| Sh3bp4 | 962 | 0.9979 | 2 | 2 | 2 | 2 | 2 | 36468.676 |
| Adgrl2 | 1487 | 0.9978 | 2 | 2 | 2 | 1 | 1 | 37595.832 |
| Cpt1b | 772 | 0.9971 | 2 | 2 | 2 | 2 | 2 | 79472.46 |
| Nap1l4 | 375 | 0.9966 | 3 | 2 | 4 | 2 | 3 | 58837.258 |
| Ndufv1 | 464 | 0.9949 | 2 | 2 | 2 | 2 | 2 | 143538.1 |
| Mcm5 | 734 | 0.9944 | 1 | 1 | 1 | 1 | 1 | 32864.016 |
| Itga1 | 1179 | 0.9942 | 2 | 2 | 2 | 1 | 1 | 68021.586 |
| Aoc3 | 765 | 0.9932 | 2 | 2 | 2 | 2 | 2 | 47923.16 |
| Cav3 | 151 | 0.9931 | 2 | 1 | 7 | 1 | 4 | 21725.443 |
| Kif3a | 701 | 0.9907 | 1 | 1 | 1 | 1 | 1 | 99137.3 |
| Park7 | 189 | 0.989 | 1 | 2 | 2 | 2 | 2 | 82981.79 |
| Krt82 | 516 | 0.9873 | 1 | 8 | 8 | 7 | 7 | 202303.36 |
| Atrx | 2476 | 0.9863 | 2 | 3 | 3 | 2 | 2 | 8707.7705 |
| Septin10 | 452 | 0.9853 | 2 | 1 | 8 | 1 | 3 | 66528.14 |
| Sestd1 | 696 | 0.9849 | 1 | 1 | 1 | 1 | 1 | 12908.924 |
| Pelp1 | 1123 | 0.9849 | 1 | 1 | 1 | 1 | 1 | 53675.586 |
| Nudcd1 | 582 | 0.9798 | 2 | 2 | 2 | 1 | 1 | 119943.13 |
| Anapc5 | 740 | 0.9796 | 1 | 2 | 2 | 2 | 2 | 15431.921 |
| Ighv1-61 | 117 | 0.9733 | 1 | 2 | 2 | 2 | 2 | 38357.887 |
| Pkp1 | 728 | 0.9732 | 1 | 1 | 1 | 1 | 1 | 77257.734 |
| 0 | 129 | 0.9728 | 1 | 2 | 2 | 2 | 2 | 286261 |
| Erp44 | 406 | 0.9719 | 1 | 2 | 2 | 2 | 2 | 45047.902 |
| Prdx3 | 257 | 0.9716 | 1 | 1 | 1 | 1 | 1 | 25811.031 |
| Fyttd1 | 317 | 0.9715 | 1 | 1 | 1 | 1 | 1 | 42056.11 |
| Cdyl | 593 | 0.9715 | 1 | 2 | 2 | 2 | 2 | 46293.7 |
| Ttc38 | 465 | 0.9698 | 1 | 2 | 2 | 2 | 2 | 23024.332 |
| Fcgr4 | 249 | 0.9687 | 1 | 1 | 1 | 1 | 1 | 19982.314 |
| 0 | 322 | 0.9674 | 1 | 3 | 3 | 1 | 1 | 92829.96 |
| Lemd3 | 921 | 0.967 | 1 | 1 | 1 | 1 | 1 | 28643.209 |
| Aars1 | 968 | 0.9667 | 1 | 1 | 1 | 1 | 1 | 134180.27 |
| Ppp1cc | 323 | 0.9659 | 4 | 1 | 12 | 1 | 6 | 39538.793 |
| Dapk1 | 1442 | 0.9659 | 1 | 1 | 1 | 1 | 1 | 7683.0713 |
| Ube2d3 | 147 | 0.9656 | 1 | 0 | 1 | 0 | 1 | 67908.85 |
| Cyp2s1 | 501 | 0.9651 | 1 | 1 | 1 | 1 | 1 | 49752.63 |
| Ift122 | 1182 | 0.961 | 1 | 1 | 1 | 1 | 1 | 36893.344 |
| Aqr | 1481 | 0.9606 | 1 | 1 | 1 | 1 | 1 | 50071.273 |
| Amy1 | 511 | 0.9605 | 1 | 0 | 1 | 0 | 1 | 49545.137 |
| Tmed9 | 235 | 0.96 | 1 | 1 | 1 | 1 | 1 | 32913.684 |
| Bcat2 | 393 | 0.9579 | 1 | 1 | 1 | 1 | 1 | 38510.254 |
| Cystm1 | 104 | 0.9579 | 1 | 1 | 1 | 1 | 1 | 70653.31 |
| Mapt | 733 | 0.9573 | 1 | 1 | 1 | 1 | 1 | 29801.107 |
| 0 | 108 | 0.9569 | 1 | 5 | 5 | 2 | 2 | 3301.3228 |
| Ybx3 | 361 | 0.9553 | 4 | 2 | 5 | 2 | 3 | 21180.895 |
| Tamalin | 392 | 0.9549 | 1 | 1 | 1 | 1 | 1 | 60667.945 |
| Lclat1 | 376 | 0.9526 | 1 | 2 | 2 | 1 | 1 | 107226.12 |
| Tmtc3 | 920 | 0.9517 | 1 | 1 | 1 | 1 | 1 | 65242.39 |
| Tspan8 | 235 | 0.9514 | 1 | 1 | 1 | 1 | 1 | 141696.31 |
| Fabp5 | 135 | 0.9508 | 1 | 2 | 2 | 1 | 1 | 27333.037 |
| Aco1 | 889 | 0.9505 | 1 | 1 | 1 | 1 | 1 | 32865.69 |
| Septin5 | 369 | 0.9504 | 2 | 1 | 3 | 1 | 2 | 17401.865 |
| Dcakd | 231 | 0.9499 | 1 | 1 | 1 | 1 | 1 | 42993.504 |
| Vps26c | 297 | 0.9477 | 1 | 1 | 1 | 1 | 1 | 21004.143 |
| Arhgef6 | 771 | 0.9474 | 4 | 1 | 7 | 1 | 4 | 43261.434 |
| Ide | 1019 | 0.9467 | 1 | 1 | 1 | 1 | 1 | 28386.232 |
| Wls | 541 | 0.946 | 1 | 2 | 2 | 2 | 2 | 30525.514 |
| Ckb | 381 | 0.9449 | 1 | 1 | 1 | 1 | 1 | 32025.824 |
| Fam120c | 1091 | 0.9393 | 1 | 1 | 1 | 1 | 1 | 21821.48 |
| Rnh1 | 456 | 0.9391 | 1 | 1 | 1 | 1 | 1 | 28371.697 |
| Nsdhl | 362 | 0.9375 | 1 | 1 | 1 | 1 | 1 | 17783.717 |
| Pdxdc1 | 787 | 0.9332 | 1 | 3 | 3 | 3 | 3 | 64833.184 |
| Psma5 | 241 | 0.9304 | 1 | 2 | 2 | 1 | 1 | 47112.605 |
| Snu13 | 128 | 0.926 | 1 | 2 | 2 | 1 | 1 | 39480.484 |
| Hp | 347 | 0.9258 | 1 | 1 | 1 | 1 | 1 | 31931.314 |
| Trappc10 | 1258 | 0.9194 | 1 | 1 | 1 | 1 | 1 | 15825.073 |
| Fam83b | 1012 | 0.9186 | 1 | 1 | 1 | 1 | 1 | 39236.547 |
| Krt84 | 603 | 0.9155 | 4 | 1 | 21 | 1 | 12 | 23919.236 |
| Anapc2 | 837 | 0.9152 | 1 | 1 | 1 | 1 | 1 | 18238.5 |
| Lamp2 | 415 | 0.9101 | 1 | 1 | 1 | 1 | 1 | 13152.948 |
| Drc12 | 202 | 0.909 | 1 | 1 | 1 | 1 | 1 | 100562.945 |
| Erbb2 | 1256 | 0.9051 | 2 | 1 | 2 | 1 | 1 | 21780.385 |
| Xrn1 | 1719 | 0.9035 | 1 | 1 | 1 | 1 | 1 | 34154.64 |
| Fcgr1 | 404 | 0.8963 | 1 | 1 | 1 | 1 | 1 | 34152.25 |
| Hsd17b7 | 334 | 0.8889 | 1 | 1 | 1 | 1 | 1 | 29052.209 |
| Rps19 | 145 | 0.8872 | 1 | 1 | 1 | 1 | 1 | 30600.398 |
| Mrpl38 | 380 | 0.8856 | 1 | 1 | 1 | 1 | 1 | 14012.502 |
| Rtkn2 | 604 | 0.8854 | 1 | 1 | 1 | 1 | 1 | 15678.284 |
| Eef1b | 225 | 0.8707 | 1 | 2 | 2 | 1 | 1 | 299176.3 |
| Ipo11 | 975 | 0.8675 | 1 | 1 | 1 | 1 | 1 | 23575.176 |
| Tusc3 | 347 | 0.8596 | 1 | 1 | 1 | 1 | 1 | 47048.68 |
| Grap | 217 | 0.8512 | 1 | 1 | 1 | 1 | 1 | 32361.455 |
| Aph1a | 265 | 0.8451 | 1 | 1 | 1 | 1 | 1 | 52007.793 |
| Ptpa | 323 | 0.836 | 1 | 1 | 1 | 1 | 1 | 20300.541 |
| Pdlim3 | 316 | 0.8271 | 1 | 1 | 1 | 1 | 1 | 56582.023 |
| Alox5 | 674 | 0.822 | 1 | 1 | 1 | 1 | 1 | 15142.672 |
| Wwc1 | 1104 | 0.8141 | 1 | 1 | 1 | 1 | 1 | 46151.996 |
| Chmp2b | 213 | 0.8015 | 1 | 1 | 1 | 1 | 1 | 135816.97 |
| Ssh2 | 1423 | 0.8002 | 1 | 1 | 1 | 1 | 1 | 22293.156 |
| Krt12 | 487 | 0.797 | 1 | 2 | 2 | 2 | 2 | 31848.957 |
| Slc2a1 | 492 | 0.7959 | 1 | 1 | 1 | 1 | 1 | 21083.168 |
| Xpc | 930 | 0.7838 | 1 | 1 | 1 | 1 | 1 | 102374.2 |
| Mrps34 | 218 | 0.777 | 1 | 1 | 1 | 1 | 1 | 11983.342 |
| Plcl1 | 1096 | 0.7762 | 1 | 0 | 1 | 0 | 1 | 154783.94 |
| Praf2 | 178 | 0.7762 | 1 | 1 | 1 | 1 | 1 | 41385.11 |
| Inpp1 | 396 | 0.7745 | 1 | 1 | 1 | 1 | 1 | 17501.113 |
| Ermp1 | 898 | 0.7649 | 1 | 1 | 1 | 1 | 1 | 47570.96 |
| Prune2 | 3084 | 0.7587 | 1 | 2 | 2 | 2 | 2 | 37409.2 |
| Rnasel | 735 | 0.7523 | 1 | 2 | 2 | 1 | 1 | 30160.941 |
| Srbd1 | 982 | 0.7495 | 1 | 1 | 1 | 1 | 1 | 71707 |
| Tbc1d17 | 645 | 0.7409 | 1 | 1 | 1 | 1 | 1 | 14697.334 |
| Cfap58 | 873 | 0.7397 | 1 | 2 | 2 | 2 | 2 | 52754.13 |
| Aldh16a1 | 802 | 0.7234 | 1 | 1 | 1 | 1 | 1 | 29999.559 |
| Dipk2a | 430 | 0.7046 | 1 | 1 | 1 | 1 | 1 | 26167.834 |
| Trio | 3102 | 0.702 | 1 | 1 | 1 | 1 | 1 | 18709.223 |
| Xirp1 | 1129 | 0.6961 | 1 | 1 | 1 | 1 | 1 | 18398.965 |
| Faah | 579 | 0.6924 | 1 | 1 | 1 | 1 | 1 | 11064.64 |
| Clint1 | 631 | 0.6863 | 1 | 1 | 1 | 1 | 1 | 18727.035 |
| Myot | 496 | 0.6782 | 1 | 2 | 2 | 2 | 2 | 80272.89 |
